# Isolation and Characterization of Cellulose from Different Fruit and Vegetable Pomaces

**DOI:** 10.3390/polym9100495

**Published:** 2017-10-09

**Authors:** Monika Szymańska-Chargot, Monika Chylińska, Karolina Gdula, Arkadiusz Kozioł, Artur Zdunek

**Affiliations:** Institute of Agrophysics, Polish Academy of Sciences, Doswiadczalna 4, 20-290 Lublin, Poland; m.chylinska@ipan.lublin.pl (M.C.); k.gdula@ipan.lublin.pl (K.G.); a.koziol@ipan.lublin.pl (A.K.); a.zdunek@ipan.lublin.pl (A.Z.)

**Keywords:** agro-industrial waste, pomace, biorefinery, cellulose structure, biomass

## Abstract

A new fractionation process was developed to achieve valorization of fruit and vegetable pomaces. The importance of the residues from fruits and vegetables is still growing; therefore; the study presents the novel route of a fractioning process for the conversion of agro-industrial biomasses, such as pomaces, into useful feedstocks with potential application in the fields of fuels, chemicals, and polymers. Hence, the biorefinery process is expected to convert them into various by-products offering a great diversity of low-cost materials. The final product of the process is the cellulose of the biofuel importance. The study presents the novel route of the fractioning process for the conversion of agro-industrial biomasses, such as pomaces, into useful feedstocks with a potential application in the fields of fuels, chemicals, and polymers. Therefore the aim of this paper was to present the novel route of the pomaces fraction and the characterization of residuals. Pomaces from apple, cucumber, carrot, and tomato were treated sequentially with water, acidic solution, alkali solution, and oxidative reagent in order to obtain fractions reach in sugars, pectic polysaccharides, hemicellulose, cellulose, and lignin. Pomaces were characterized by dry matter content, neutral detergent solubles, hemicellulose, cellulose, and lignin. Obtained fractions were characterized by the content of pectins expressed as galacturonic acid equivalent and hemicelluloses expressed as a xyloglucan equivalent. The last fraction and residue was cellulose characterized by crystallinity degree by X-ray diffractometer (XRD), microfibril diameter by atomic force microscope (AFM), and overall morphology by scanning electron microscope (SEM). The hemicelluloses content was similar in all pomaces. Moreover, all the materials were characterized by the high pectins level in extracts evaluated as galacturonic acid content. The lignins content compared with other plant biomasses was on a very low level. The cellulose fraction was the highest in cucumber pomace. The cellulose fraction was characterized by crystallinity degree, microfibril diameter, and overall morphology. Isolated cellulose had a very fine structure with relatively high crystalline index but small crystallites.

## 1. Introduction

The food European sector generates about 250 million tons per year of by-products and waste, of which around 10% remain after processing of fruit and vegetables [1]. Moreover, 30–50% of those wastes and by-products come from fruit and vegetable juice production [2]. The pomaces are partially used as feed for livestock, fertilizer, or as a source of pectic polysaccharides or general dietary fiber, but still a large part of them are usually discarded, causing environmental pollution, but some of them are not [3,4,5,6,7]. Owing to rapidly expanding global demand on manufacturing processes and final products employing minimal or no environmental risk, fruit and vegetable waste processing has been the subject of significant research during the last years [8,9]. Various by-products are available offering a great diversity of low-cost raw materials. Biorefinery processes are expected to convert them into new added-value products such as, for instance, dietary fiber (non-starch polysaccharides and lignin), oligosaccharides, and polyphenols. The main residue after fruit and vegetable processing is pomace containing pulp, peel, seeds, and stem. A significant amount takes non-starch polysaccharides (35–60% dietary fiber) with a high amount of both insoluble and soluble fibers. The main constituents are pectins (1.50–13.40%), cellulose (7.20–43.60%), hemicelluloses (4.26–33.50%), lignins (15.30–69.40%), and gums [10,11]. Cellulose, a linear polymer of d-glucose, forms microfibrils that have a stiff, ordered structure, which is responsible for strength and the resistance to the degradation of this polymer [12]. Hemicelluloses are a group of polysaccharides with a structure that depends on the source, i.e., the type of plant and plant tissue. Xyloglucan is the most abundant hemicellulose, with the same d-glucose backbone as cellulose. Lignin is an amorphous complex cross-linked phenolic polymer of phenylpropane units (*p*-coumaryl, coniferyl and sinapyl alcohol), held together by different linkages [13]. The conversion of lignocellulosic biomass is strong and dependent not only on the biochemical composition but also on the structural features of plant tissue [14,15]. Hence, the pretreatment of the lignocellulosic biomass is an essential step to increase cellulose and hemicelluloses accessibility in the biorefinery process [16,17].

In this study, pomaces, after the most often processed fruits and vegetables in Europe such as apple, carrot, tomato and cucumber, were used [18]. Apple (*Malus* sp.) is the most processed fruit that generates high amount of wastes. In 2013 global production of this fruit reached 67.9 million tons, which produced annually around 12 million tons of wastes [10,19]. Tomato (*Solanum lycopersicum* L.) is a widely consumed vegetable crop with a world production of over 170 million tons in 2014 [20]. Tomatoes are processed yearly to produce tomato juice, paste, purée, ketchup, sauce, and salsa, resulting in generation of large quantities of wastes [21]. Tomato pomace obtained after pressing is composed of around 33% seed, 27% skin, and 40% pulp [22]. Another lignocellulosic material produced in large quantities during the process of juice extraction in the industry is carrot pomace. Although this agricultural residue may be used as an animal feed, it is usually discarded as waste [23]. Carrot pomace is composed of 28% cellulose, 2.1% pectin, 6.7% hemicellulose, and 17.5% lignin on a dry weight basis [11]. Cucumber is processed for preservation (pickles) or as a fresh-cuts for “ready-to-eat” vegetables. The main waste of cucumber processing is either its peel or whole slices, which comprise around 12% of processed cucumbers [24,25].

The fractions of pomaces such as pectins, hemicelluloses, and cellulose can have broad applications in the food, as well as in the non-food (pharmaceutical, cosmetics, and polymers), industry [26,27]. Due to their biodegradability, biocompatibility, edibility, and versatile chemical and physical properties, each of the polysaccharides plays an important role in designing functional food, new biomaterials, or carriers for drugs or bioactive substances [28]. Pectins are broadly used as a thickener, a gelling and texture agent in dairy or baking, and carriers for drug delivery systems in the cosmetic and pharmaceutical industries [29,30,31,32]. Hemicelluloses are applicable as gel materials, films, coatings, and adhesives in technical and pharmaceutical fields [33,34]. In food products, hemicelluloses are used as stabilizing, viscosity-enhancing, and gelling agents. One of the most common hemicelluloses, xyloglucan, is able to interact with cellulose and can be used as surface treatment for cellulosic materials in dyeing. Therefore, xyloglucan has found an application in the textile industry, cosmetics, wet-end additives, papermaking, and in-situ-gelling preparations [35]. Cellulose, in various forms, can be used in food applications, i.e., as a fat substitute, texturizer, emulsifier, and bulking agent in low-caloric foods, while also playing an important role in the production of biofuels, designing new biomaterials and nanocomposites mainly in medicine, pharmacy, and the packaging industry [36,37].

The conventional fractionation method of pectins from fruit pomace consists of treating the raw materials with hot mineral acids such as hydrochloric, sulfuric, and nitric acids at a pH range of 1.5–3.0 [6,15,30]. pH and temperature extractions influence the yield and quality of pectins. At an industrial scale, acid- or base-treated pomace is subjected to subsequent extraction with diluted acid. These conventional techniques are superseded by ultrasound- or microwave-assisted extraction techniques [38,39]. Recently, pectins have been isolated in more environmentally friendly ways by protein precipitation based on charge neutralization of pectin regions using sodium caseinate [9]. The conventional extraction process of xyloglucans is achieved by soaking and mixing the pomace in alkaline solutions such as sodium hydroxide (NaOH), potassium hydroxide (KOH), and lime solutions (Ca(OH)_2_) [38,39,40]. The alkali concentration and the duration of extraction have been determined as the main factors influencing the extraction yield. As in the case of pectins extraction, the ultrasound waves support the alkaline extraction of hemicelluloses [9]. The isolation of cellulose from fruit pomace generally consists of combined alkaline extraction in the presence of hydrogen peroxide or sodium hypochlorite. Hereby, the hydrogel form of microcrystalline cellulose is obtained [9,10].

In the case of pomaces, many studies report the feasibility of polysaccharides extraction at large scale [41]. However, the fraction of the core components, i.e., cellulose, hemicelluloses, and pectins, will generate higher added value from fruit and vegetable by-products. On the other hand, the feasibility of using any lignocellulosic feedstock as a bioresource depends on its cell wall saccharification potential. In turn, this depends on the accessibility and susceptibility of cell wall polysaccharides to hydrolytic enzymes, and the chemical composition of the plant cell wall, which offers some barriers that hinder this task. Therefore, the objective of the present study was to develop a path of pomaces valorization and, indirectly, cellulose isolation. 

The chemical method was chosen in order to fraction the biomass components such as lignins, hemicelluloses, and pectins and, finally, for the isolation of cellulose fibers. Moreover, the yield of polysaccharides fractions was estimated. The further purpose was the characterization of the structure of isolated cellulose microfibrils from fruit and vegetable pomaces. The obtained residues show potential as the source of biomass for biofuel conversion, but also as a source of biopolymers such as pectins, hemicelluloses, and cellulose to be used for biomaterials production or in the food sector. In this way, it will contribute to the recent and innovative implementation of the biorefinery concept applied to food wastes for optional use and new-value creation.

## 2. Experimental Section

### 2.1. Raw Material

Four different pomaces from apple (*Malus domestica* Borkh.), tomato (*Solanum lycopersicum* L.) cucumber (*Cucumis* L.), and carrot (*Daucus carota* L.) were used. The fresh fruits and vegetables were bought in the local grocery shop. The pomaces were prepared in a de-pulping machine with a double screw shredder (Twin Gear Juice Extractor, Green Star Elite GSE-5000, Anaheim, CA, USA) and contained fragments of pulp, skin, seeds, and stems.

### 2.2. Fractioning of Plant Biomass

**Figure 1:**
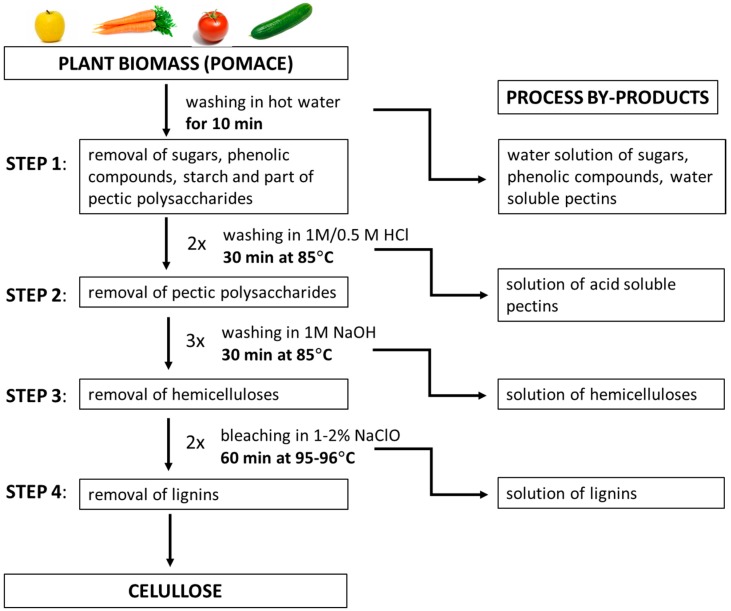
The flow chart of pomace fractioning process (patent application) [42].

The fractioning process of plant biomass is presented in Figure 1. Briefly, approximately 500 g of each pomace was taken and placed in hot water (3 L). The pomace was boiled for 10 min and filtered after that time. In this step, sugars, phenolic compounds, and part of water soluble polysaccharides were removed. Afterwards, the 3 L of 1 M hydrochloric acid solution (HCl) was added to the obtained residue and stirred for 30 min at 85 °C; subsequently, the residue was filtered. This step was repeated with 0.5 M HCl solution. Through the acid treatment, the pectic polysaccharides were removed. Thereafter, the residue was stirred in 3 L of 1 M sodium hydroxide solution (NaOH) for 30 min at 85 °C; subsequently, the residue was filtered. This step was repeated threefold. Through the alkali treatment, the hemicelluloses were removed. The next step involved the residue bleaching with 1–2% sodium hypochlorite solution for 60 min at 95–96 °C. This stage was repeated twice. The resulting precipitate was cellulose, which was washed several times with hot deionized water until a neutral pH of the filtrate was obtained.

### 2.3. Characterization of Plant Material

To obtain dry matter content of each pomace (approximately 3 g), sample was dried (SUP-30W, Wamed, Warsaw, Poland) at 105 °C to constant mass. Dry matter content (DM) was calculated as DM = (*m*_2_/*m*_1_) × 100, where m_1_ was the mass of the fresh sample and *m*_2_—mass of the dried sample.

Van Soest [43] analysis, with some modifications, was used for cellulose, hemicelluloses, and lignin determination [3,44,45]. This method enables separation of plant cell wall fractions, and thus dietary fiber fractions, by using two detergents: a neutral detergent—ND solution (sodium dodecyl sulfate, EDTA, pH 7.0) and an acidic detergent—AD solution (cetyltrimethyl ammonium bromide in 1 N H_2_SO_4_). The neutral detergent removes pectic polysaccharides, phenolic compounds, proteins, and sugars. Then, in the second step of extraction acid, detergent removes hemicelluloses. Subsequently, the cellulose is solubilized by 72% sulfuric acid. Three replicates (each approximately 0.1 g) from every pomace sample were taken and placed into glass crucible for Van Soest analysis. Termogravimetric analysis was performed with crude fiber extractor FIWE 3 (Velp Scientifica, Velate MB, Italy). Samples ware boiled in ND solution (100 mL) for 1 h, then washed by hot water and acetone and finally dried at 105 °C overnight giving NDF fraction (*m*_NDF_). In order to extract lignocellulosic yield, the procedure was analogous, except that samples were boiled in AD solution giving ADF fraction (*m*_ADF_). Finally, 20 mL of sulfuric acid solution was added to the residue and the extraction of cellulose was conducted for three hours. After, the sample was extensively washed out several times with hot water and finally by acetone. ADL fraction (*m*_ADL_) was received.

### 2.4. The Hemicelluloses Yield Was Estimated

(1) $$H\;[g/100g]=\frac{m_{\mathrm{NDF}}-m_{\mathrm{ADF}}}{m_{\mathrm{SAMPLE}}}\cdot 100$$

cellulose yield:

(2) $$C[g/100g]=\frac{m_{\mathrm{ADF}}-m_{\mathrm{ADL}}}{m_{\mathrm{SAMPLE}}}\cdot 100$$

and lignins yield:

(3) $$L[g/100g]=\frac{m_{\mathrm{ADL}}}{m_{\mathrm{SAMPLE}}}\cdot 100$$

The cellulose (C%), hemicellulose (H%), and lignin (L%) content is expressed as an g/100 g of dry pomace fraction (m_sample_—weight of dry pomace). The content of natural detergent fibre (NDF) composed of overall hemicellulose, cellulose, and lignin was also calculated:

(4) $$\mathrm{NDF}\;[g/100g]=\frac{m_{\mathrm{NDF}}}{m_{\mathrm{SAMPLE}}}\cdot 100$$

Considering the potential use of supernatants produced in the process of the cellulose isolation, the investigation of pectins and hemicelluloses content in pomaces was carried out.

First, the pomaces were purified from sugars using hot alcohol insoluble solids method with a few modifications, as it was described before [44,46,47,48]. The purpose of purification was to avoid influence of free sugars on the pectins and hemicelluloses determination. The obtained residues, namely cell wall material (CWM), were then introduced to sequential extraction as it was described previously [3,44,49,50]. The three pectic fractions were obtained: water soluble pectins (WSP), chelator soluble pectins (CSP), and dilute alkali soluble pectins (DASP). The fraction of hemicelluloses was extracted with KOH solutions (0.5, 1, and 4 M) with addition of 10 mM sodium borohydride. Supernatants of all KOH fractions contained hemicelluloses were collected together.

The determination of pectin content in pectic fractions was performed with Automated Wet Chemistry Analyzer, known as the Continuous Flow Analyzer (San++, Skalar, Analytical, Breda, The Netherlands). This is an automated procedure for colorimetric determination of galacturonic acid based on total decomposition of pectin sample in acidic medium (sulfuric acid). The obtained products are transformed to furfural derivatives, which, when reacting with 3-phenyl phenol, form a coloured dye, with which absorbance is measured at 530 nm [44,51]. The pectins content is expressed as galacturonic acid (GalA) content in mass of samples’ CWM (mg/g CWM).

The carbohydrate (hemicelluloses) content in KOH fractions was determined by the Sulfuric Acid–UV method as follows [52]. A 1 mL aliquot of carbohydrate solution was mixed with 3 mL of concentrated sulfuric acid in a test tube and vortexed for 30 s. The temperature of the mixture rises rapidly within 10–15 s after addition of sulfuric acid. Then, the solution was cooled in ice bath for 2 min to bring it to room temperature. Finally, UV light absorption at 315 nm was measured using UV spectrophotometer. Xyloglucan from tamarind (Megazyme, Brey, Ireland) was used as a standard for calibration curve preparation and results were expressed as xyloglucan equivalents in mass of samples’ CWM (mg/g CWM).

### 2.5. Spectroscopic Investigation of Cellulose

The Raman and FT-IR spectra of isolated cellulose were recorded in order to evaluate the purity of cellulose as well as the structure of cellulose.

The FT-IR spectra of celluloses isolated from pomaces were collected with the usage of a Nicolet 6700 FT-IR spectrometer (Thermo Scientific, Waltham, MA, USA) with the Smart iTR ATR sampling accessory. Each material were placed on ATR crystal as a powder. All spectra were recorded in the range of 4000–650 cm^−1^. For each material, five samples under the same conditions were examined and 200 scans were taken per sample. All spectra were measured at a spectral resolution of 4 cm^−1^ and normalized at 1030 cm^−1^. The presented FT-IR spectra are average value of respective single spectra.

The FT-Raman spectra were obtained with an FT-Raman module (NXR FT Raman) for a Nicolet 6700 FT-IR bench using an InGaAs detector and CaF_2_ beam splitter (Thermo Scientific, Madison, WI, USA). The samples were placed in stainless cube and illuminated using Nd:YAG excitation laser operating at 1064 nm. The maximum laser power was 1 W. The spectra were recorded over the range of 3500–150 cm^−1^, and each spectrum was an average of 256 scans at 8 cm^−1^ resolution. The spectra were normalized against a band at 1095 cm^−1^. The analyzed spectra were averaged over five registered spectra.

The spectra were visualized using ORIGIN (version 8.5 PRO, OriginLab Corporation, Northampton, MA, USA). The reference spectrum of Avicel microcrystalline cellulose (Avicel PH101, 50 µm, α-cellulose obtained from wood pulp, FMC Biopolymer, Brussels, Belgium) was also recorded.

### 2.6. Scanning Electron Microscopy (SEM)

Morphology of cellulose was examined by scanning electron microscope (SEM, Quanta 3D FEG, FEI Phenom Word, Eindhoven, The Netherlands) operating at 20 kV in high vacuum conditions. The samples were prepared as the water suspension in 0.1 wt % concentration. Prior to SEM observation, the drop of samples were applied on aluminum stage covered by the carbon tape and left to air dry (the room was equipped in HEPA filter). Then, the samples were coated with an ultrathin layer of palladium/gold layer (Pd/Au) in an ion sputtering machine (Quorum Techn, Polaron SC7640/CA7625, Laughton, UK). The ETD (SE) detector was used.

### 2.7. AFM Images and Microfibrils Diameter Measurement

The cellulose was suspended in ultrapure water (Millipore) to obtain the 0.1 wt % concentration. Then, suspension was deposited onto a microscopic slide and dried in room condition at 23 °C for 24 h. The samples were kept in a desiccator before AFM observations. Bioscope Catalyst II, supported with a Nanoscope V controller (Bruker, Billerica, MA, USA), was used for imaging in the semiautomatic tapping mode. A silicon nitride cantilever (Bruker) with nominal radius of pyramidal tip 2 nm, spring constant of 0.4 N·m^−1^, and resonance frequency of 70 kHz was used. The experiment was performed in ambient air at room temperature of about 20–22 °C and a relative humidity (RH) of 26–30%. Scan area 4 μm^2^ (aspect ratio 1:1) and image resolution 512 × 512 points were set. The scan rate 0.5 Hz was maintained for obtaining appropriate image quality. For each sample, around 12 images from various regions were collected.

Raw height images of cellulosic fibrils have been pre-processed in SPIP 6.2.0 software (Image Metrology, Hørsholm, Denmark). For each of images, the plane correction included global bow removal with polynomial fit of the third order; also, 3 × 3 median filter was performed. Thereafter, diameters of individual or parallel connected fibrils were evaluated manually using images from the peak force error channel. For each sample, the amount of 1015–1038 values was collected from randomly selected fibrils.

### 2.8. X-ray Diffractometry (XRD)

Degree of crystallinity cellulose isolated form pomaces was determined by means of the X-ray diffraction method. The X-ray diffractometer Empyrean (PANalytical, Almelo, The Netherlands) was used. Samples were scanned with Cu Kα radiation (λ = 0.15418 nm). The parameters of the working lamp were as follows: U = 40 kV, I = 25 mA. The intensity of reflections was measured over the angular 5–90° 2θ with step intervals of 0.05°. The duration of the reflection count was 10 s. On the basis of registered measurements, a mathematical model describing the relationship between intensity and 2θ was developed. The degree of crystallinity was calculated twofold: one according to the general equation given by Wunderlich (1973) [53], and second developed by Segal et al. (1962) [54] for cellulose:

(5) $$CI_{W}=\frac{kI_{C\;}}{I_{C}-I_{A}}\cdot 100\%$$

where CI_W_—the degree of crystallinity determined by Wunderlich method; I_C_—area under the reflex from the crystalline phase; I_A_—spectrum area of the amorphous phase; and *k*—proportionality factor, including polarization and Lorenz effect (Thompson–Lorenz factor), temperature correction, and differences between X-ray density of amorphous phase [46].

(6) $$CI_{S}=\frac{I_{002}-I_{\mathrm{am}}}{I_{002}}\cdot 100\%$$

where CI_S_—degree of crystallinity determined by Segal method; I_002_—the intensity value for the crystalline cellulose (2θ = 22.5°); and I_am_—the intensity value for the amorphous cellulose (2θ = 18°) [55]. The crystallites size was calculated using modified Scherrer equation [56,57]:

(7) $$d=\frac{0.9\cdot \lambda }{\sqrt{\left({\mathrm{FWHM}}^{2}-b^{2}\right)}\cdot \cos\theta }$$

where λ—the X-ray wavelength (0.1542 nm); FWHM—the full width at half maximum of the diffraction band; *b*—instrumental factor (0.1489); and θ—the Bragg angle corresponding to the (002) plane.

## 3. Results

### 3.1. The Composition of Plant Pomaces

The content of individual components in the investigated pomaces such as hemicellulose, lignins, and cellulose were analyzed. The percentage of components in samples was determined by standard raw fiber determination method [43]. The result of this determination is expressed in g/100 g of dry pomaces content of hemicellulose (H%), cellulose (C%), and lignin (L%) (Table 1). The percentage of crude fiber NDF fraction (hemicelluloses, celluloses, and lignin) in the dry matter of selected plant samples was 18.23% for carrot, 19.77% for tomato, 24.97% for cucumber, and 17.22% for apple pomaces. The average content of cellulose was oscillating around 10% with highest content for cucumber pomace (16.13%) and the lowest for tomato (8.60%) and apple (8.81%) pomaces. The lignin content was the highest in the case of tomato (5.85%) and the lowest for carrot (2.50%) and apple (2.98%) pomaces, while for cucumber it was around 4.51%. The hemicellulose content was on average 4–5%.

In addition, polygalacturonic acid and hemicellulose fractions isolated from pomaces were analyzed. Water soluble pectin (WSP), calcium chelator soluble pectins (CSP), and diluted alkali soluble pectins (DASP) fractions were obtained, as well as a fraction of hemicellulose extracted with various concentrations of KOH solutions. In pectin fractions, galacturonic acid content was determined, while in the fraction extracted with KOH, the hemicelluloses content as equivalent of xyloglucan was determined (Table 2).

The pectic fractions isolated from carrot pomace were the richest in GalA total content (186.48 mg of GalA in g of CWM). Moreover, the carrot pomace was the richest in DASP fraction (130.49 mg of GalA in g of CWM), which mainly consists of the branched pectins. Whereas, the lowest total content of GalA was obtained for tomato pomace (135.87 mg of GalA in g of CWM), with the highest content of GalA in WSP fraction (60.57 mg of GalA in g of CWM) mainly consisting of linear homogalacturonan [51,58]. The total content of GalA in pectic fractions obtained for apple and cucumber pomaces were on the similar level around 150 mg of GalA in g of CWM. The cucumber pomace was the richest in CSP fraction (71.84 mg of GalA in g of CWM); however, the pectins level in this fraction was quite similar for all pomaces. The hemicelluloses content determined in KOH fraction obtained for all pomaces varied from 56.24 mg (cucumber pomace) to 99.88 mg of Xyl in 1 g of CWM (carrot pomace).

### 3.2. Cellulose Morphology Analysis: SEM and AFM

**Figure 2:**
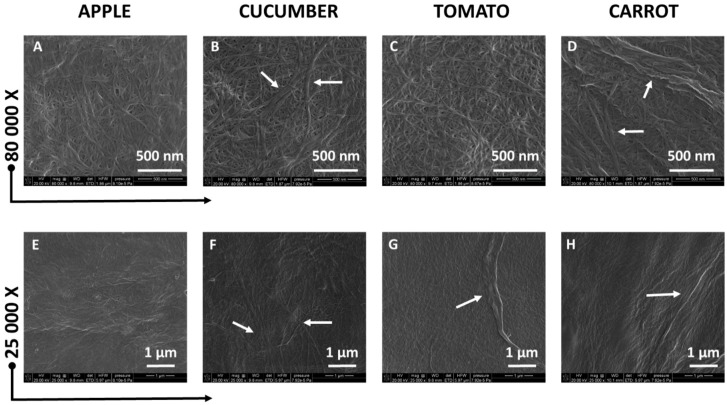
SEM micrographs presenting the cellulose isolated from apple (**A**,**E**), cucumber (**B**,**F**), tomato (**C**,**G**), and carrot (**D**,**H**) pomaces. White arrows mark the cellulose macrofibrils presence.

Figure 2A–H presents SEM images of cellulose isolated from pomaces under two magnifications. The cellulose suspension of 0.1 wt % concentration after drying on the carbo tape formed a film. Moreover, the differences in morphology of cellulose isolated from different pomaces can be visible. Micrographs obtained with high magnifications (Figure 2A–D) showed uniform distribution of cellulose microfibrils with comparable thickness. However, in the case of cellulose isolated from cucumber and carrot pomaces, also cellulose macrofibrils are visible (Figure 2B,D). SEM micrographs with lower magnification confirmed the homogeneity of microfibrils distribution and presence of macrofibrils (Figure 2E–H).

**Figure 3:**
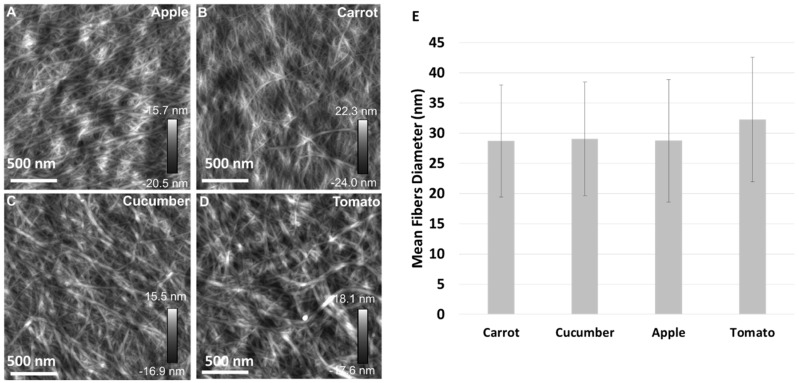
Representative atomic force microscope (AFM) height images of cellulose isolated from apple (**A**), carrot (**B**), cucumber (**C**), and tomato (**D**) pomaces and mean microfibril diameters of individual samples (**E**).

**Figure 4:**
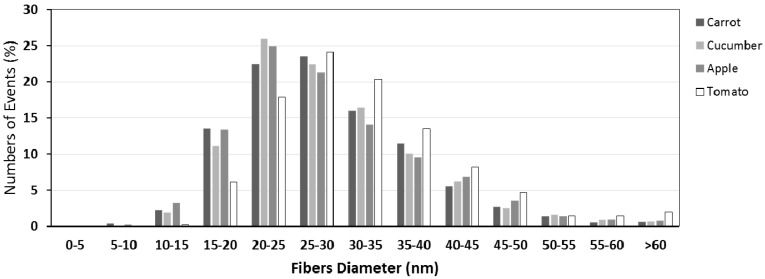
Histograms of cellulose microfibrils diameter distribution of cellulose isolated from apple, carrot, cucumber, and tomato pomaces.

The diameter of cellulose microfibrils was evaluated by AFM. The height images of cellulose isolated from pomaces are presented (Figure 3A–D). In contrast to SEM micrographs, the macrofibrils weren’t visible on AFM images. The microfibril mean diameter (Figure 3E), as well as distribution of microfibril diameters (Figure 4), were estimated. Difference among mean diameters of cellulose microfibrils isolated from pomaces were negligible. Their thickness was, on average, 28.68 (±9.27) nm for carrot pomace, 29.03 (±9.43) nm for cucumber pomace, 28.73 (±10.17) nm for apple pomace, and 32.24 (±10.35) nm for tomato pomace. In general, the cellulose microfibril diameter depends on its origin. The diameter distribution of microfibrils was similar for cellulose isolated from carrot, cucumber, and apple pomaces with the largest diameter fraction (more than 45% of estimated microfibrils) between 20–30 nm, while in the case of cellulose microfibrils isolated from tomato, the distribution maximum (more than 45% of estimated microfibrils) is shifted to the thicker fractions of 25–35 nm. What is more, for the tomato pomace cellulose 2% of the microfibrils were those with diameter larger than 60 nm compared with ca. 0.6% in the other cases. The finest microfibrils were obtained for carrot and apple pomaces’ celluloses—around 3% of microfibrils were thinner than 15 nm.

### 3.3. Cellulose Structure

**Figure 5:**
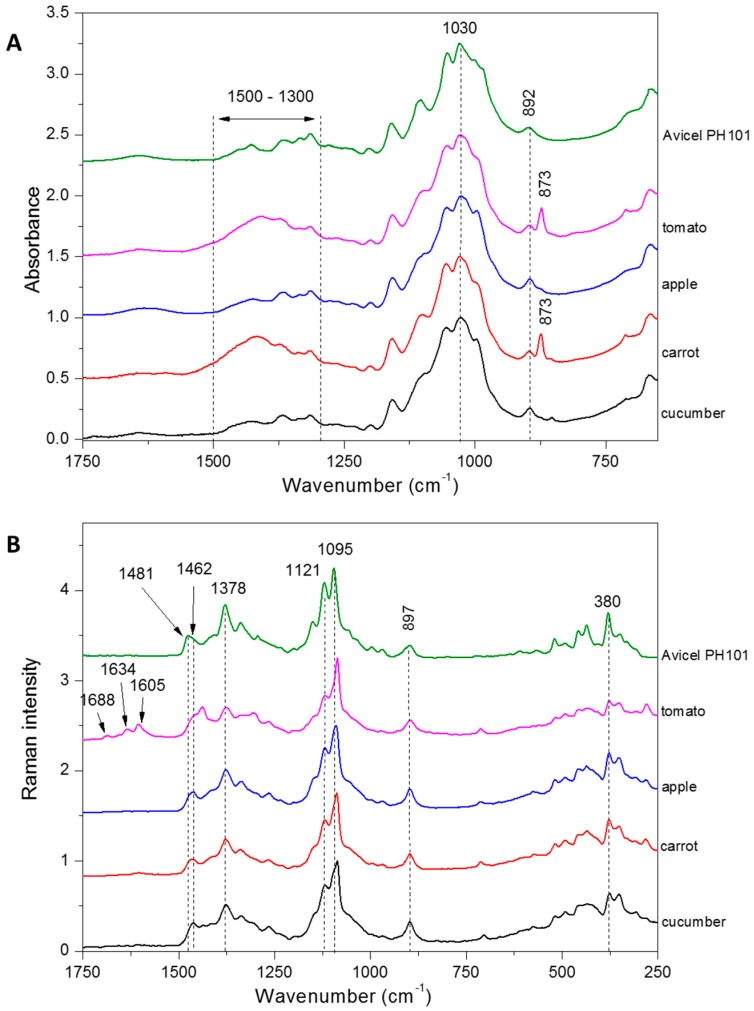
FTIR (**A**) and Raman (**B**) spectra of cellulose isolated from tomato, apple, carrot, and cucumber pomaces. The Avicel PH101 is presented here as an example of pure microcrystalline cellulose with well-defined structure. FT IR spectra are presented in the range of 1750–650 cm^−1^ and Raman are presented in the range of 1750–650 cm^−1^. The most characteristic bands are highlighted on spectra with dashed line.

The FTIR and Raman spectra were obtained for cellulose isolated from selected pomaces (Figure 5). Microcrystalline cellulose (PH 101, Avicel) was used as a reference sample. Both types of spectra can serve as a determinant of the purification degree of the samples. FTIR spectrum of isolated celluloses showed characteristic bands at 1030 cm^−1^ and 892 cm^−1^ assigned to C–O stretching vibration and the glycosidic-C_1_H deformation, respectively [59]. In the case of FTIR spectra for cellulose isolated from tomato and carrot extracts, an additional 873 cm^−1^ band is visible, along with a significant broadening of bands in the range of 1300–1500 cm^−1^ (Figure 5A). The region of 900–800 cm^−1^ (C–H out-of-plane bending) may be due to the aromatic compounds vibrations as phenolic compounds [59,60]. Whereas, the broadening in region 1300–1500 cm^−1^ can be associated with trace of proteins [59].

In the Raman spectra, typical and characteristic cellulose bands can be visible in samples from different pomaces (Figure 5B). The band around 380 cm^−1^ is assigned as bending vibration of CCC and is the ring deformational mode; 897 cm^−1^ bending vibration of HCC and HCO, 1121 and 1095 cm^−1^, are assigned to the symmetric and asymmetric stretching mode of COC in the glycosidic bond, and 1378 cm^−1^ bending vibration of groups HOC, HCO, and HCC [12,61,62]. Ones of the most characteristic bands for cellulose molecules are assigned as bending vibration of HCH and occur around 1481 and 1462 cm^−1^. The shift from 1481 cm^−1^ (highly crystalline cellulose) to 1462 cm^−1^ (amorphous cellulose), and also change in proportions of these two bands, can serve as an indicator of crystalline structure of cellulose [63]. When comparing the spectra of celluloses isolated from pomaces with microcrystalline cellulose (Avicel), this visible shift could be observed. In the case of microcrystalline cellulose Avicel PH101, which has a crystallinity degree of around 53.8%, the band was shifted to 1481 cm^−1^, but for celluloses isolated from pomaces this band was shifted to 1462 cm^−1^, which indicates that celluloses from pomaces are less crystalline than Avicel cellulose [12]. This band is mostly shifted to 1455 cm^−1^ in the case of tomato cellulose, which can be evidence of its lower crystallinity index. Also, additional bands were observed in the range of 1600 to 1700 cm^−1^ in spectrum of cellulose isolated from tomato pomace (Figure 5B). The bands in this range are evidence of lignin presence, which probably has not been removed completely during the bleaching process [64,65]. This means that for pomaces with high lignin content, as it was in the case of tomato pomace, it is necessary to extend the bleaching time of cellulose. In addition, it should be noted that for the rest of the samples these bands are absent, which indicates high purity of isolated cellulose.

The crystallinity and crystallites’ average size of isolated celluloses was determined using XRD diffractograms using two methods: one created by Wunderlich and the other by Segal. In the case of cellulose isolated from pomaces, the Segal crystallinity index ranged from 48.97% for tomato cellulose to as high as 68.73% for carrot cellulose. Whereas, for apple and cucumber cellulose the crystallinity index was 51.34% and 53.61%, respectively. The crystallinity indexes determined by Wunderlich method were lower than Segal crystallinity index, but the trend was the same: the highest crystallinity degree was obtained for cellulose isolated from carrot pomace (56.51%) and the lowest for tomato cellulose (34.29%). The determined crystallites’ size was around 2–3 nm. Even though the differences between crystalline structures of celluloses were minimal, the differences in crystallites size were obtain. Also, the largest crystallites were obtained for carrot, while more crystalline cellulose and the smallest crystallites were obtained for tomato less crystalline cellulose.

## 4. Discussion

The composition of lignocellulosic biomass has tremendous effect on the efficiency of cellulose hydrolysis and bioconversion. That’s why the selection of biomass pretreatment, as well as knowledge of biomass composition itself, is important [66]. Among other main factors limiting biomass conversion is crystallinity degree of cellulose, cellulose covering by hemicellulose, and lignin content [66]. Therefore, the pomaces were characterized in terms of neutral detergent fibre (NDF), hemicelluloses, cellulose and lignins content, as well as the content of pectins and hemicelluloses in extracts from the pomaces. The greatest contribution of fibrous fraction (NDF) was in the case of cucumber pomace. The hemicelluloses content was similar in all pomaces. However, determination of hemicelluloses in KOH extract showed that the highest value of extractable hemicelluloses was in carrot and the lowest in cucumber pomace. Whereas, all the materials were characterized by the high pectins level in extracts (evaluated as galacturonic acid content). Moreover, above results show the direction in possible use of the extraction solutions which are particularly rich in valuable polysaccharides such as pectins and hemicelluloses. The lignins content was the highest in tomato pomace due to high content of peels and seeds in that material. However, it can be concluded that comparing with other plant biomass, such as wood chips and pulp or fibrous plants, the overall lignins content in examined pomaces was on a very low level. The composition of other lignocellulosic sources such as cereals straws (wheat, barley, rice), corn stover, banana peels, or pineapple leaf fibers differs from values obtained for pomaces and contains from 38% to 82% of cellulose, 7–38% of lignins, and 6–38% of hemicelluloses [67,68]. Even though the pomaces contained relatively small amount of cellulose, they were easily accessible due to the high content of extractives such as pectins, sugar, phenolic compounds, and low content of hemicelluloses and lignin. The cellulose fraction was the highest in cucumber pomace and in the rest pomaces it was lower or on a similar level. Taking into consideration the pomaces as a source of cellulose, the most promising are the apple and carrot pomaces, which were characterized also by the lowest lignins content. It simplifies the cellulose isolation process and also has impact on cellulose accessibility to enzymatic hydrolysis used during bioethanol production. Moreover, isolated cellulose had very fine structure with relatively high crystalline index but small crystallites. In general, the cellulose microfibril diameter depends on its origin. For example, bacterial and cotton celluloses are very thin with diameter from 5 to 10 nm. Also, primary cell wall cellulose is thin, with microfibril diameter from parenchyma cell walls of about 1.5–3 nm [67]. Whereas, it was reported that the wood delignified cellulose is 20–40 µm in diameter [69]. The diameter distribution of microfibrils was similar for cellulose isolated from carrot, cucumber, and apple pomaces with average value of 29 nm. Meanwhile, in the case of cellulose microfibrils isolated from tomato, the average microfibrils diameter was around 32 nm. The crystallinity and crystallites average size of isolated celluloses was determined using XRD diffractograms. The crystallinity degree of algal or bacterial cellulose reaches values of 65% and even higher. Whereas, for cellulose isolated from fibrous plants or wood pulp, it ranges from 40–60% [69]. In the case of cellulose isolated from pomaces, the crystallinity degree varied from 34% (tomato) to 56% (carrot). Whereas, the determined crystallites’ size was around 2–3 nm and comparable with the values obtained for cellulose isolated from wood or vegetal fibres [56]. Those results support the possible use of cellulose as a component of biomaterials (reinforcing agent) or its further processing to obtain nanocrystalline or nanofibrous cellulose.

Here mild acid-alkali conditions were used for the fractioning process of biomass. This approach ensures the minimum degradation of valuable substances contained in pomaces such as pectic and hemicellulosic polysaccharides [70]. Also, low-temperature process was chosen contrary to high-temperature hydrothermal processes [15]. In the case of pomaces, the polysaccharides are weakly bonded among each other and are packed in less structured tissue compared with wood or grass biomass. Hence, the low temperature is sufficient enough to extract pectic or hemicellulosic polysaccharides. Moreover, it was reported that low temperature calcium hydroxide treatment generates an additional effect regarding methane production from biomass [71].

## 5. Conclusions

The concept of the conversion of fruit and vegetable pomaces as an example of agro-industrial residues into valuable by-products is presented. To the best of our knowledge, this is the first comprehensive study of apple, carrot, cucumber, and tomato pomaces for valorization purposes. Fruit and vegetable pomaces can be used not only as a substrate for bioconversion or a feed for livestock but also as a source of valuable by-products which could be reused as food additives or in biomaterials. Investigated pomaces were relatively low in lignin, which has allowed for easier access to other components such as pectins, hemicelluloses, or cellulose. In consequence, the biomass fractioning process did not require a long processing time or toxic chemicals, which also reduced the economic and environmental costs of pomace components isolation.

## Figures and Tables

**Table 1:** Dry matter, neutral detergent fibre (NDF), lignin, hemicellulose, and cellulose content in pomaces.

POMACE	Dry Matter Content	Cellulose	Hemicellulose	Lignin	NDF
	%	g/100 g DRY POMACE	g/100 g DRY POMACE	g/100 g DRY POMACE	g/100 g DRY POMACE
Carrot	13.10 (±0.17)	10.01 (±0.20)	5.73 (±0.27)	2.50 (±0.19)	18.24 (±0.28)
Tomato	6.03 (±0.22)	8.60 (±0.34)	5.33 (±0.92)	5.85 (±0.32)	19.77 (±1.52)
Cucumber	5.57 (±0.05)	16.13 (±0.90)	4.33 (±1.32)	4.51 (±0.54)	24.98 (±0.52)
Apple	17.84 (±0.50)	8.81 (±0.51)	5.44 (±0.49)	2.98 (±0.67)	17.22 (±1.21)

The standard deviation is presented in parentheses.

**Table 2:** The content of polysaccharide fractions isolated from the pomaces. TOTAL GalA is counted as sum of WSP, CSP, and DASP fractions GalA content. WSP—water soluble pectins fraction; CSP—chelator soluble pectins fraction; DASP—diluted alkali soluble pectins fractio; GalA—galacturonic acid; Xyl—xyloglucan; CWM—cell wall material isolated from pomaces.

POMACE	Polysaccharide Fraction				
	KOH	WSP	CSP	DASP	TOTAL
	mg Xyl/g CWM	mg GalA/g CWM	mg GalA/g CWM	mg GalA/g CWM	mg GalA/g CWM
**Carrot**	99.88 (±0.49)	12.21 (±0.15)	43.77 (±0.60)	130.49 (±0.59)	186.48 (±1.34)
**Tomato**	89.64 (±1.60)	60.57 (±0.63)	67.02 (±0.44)	8.29 (±0.08)	135.87 (±1.15)
**Cucumber**	56.24 (±1.94)	16.25 (±0.91)	71.84 (±0.73)	71.68 (±1.75)	159.76 (±3.39)
**Apple**	70.93 (±1.34)	22.40 (±1.26)	51.29 (±0.54)	78.41 (±0.74)	152.10 (±2.55)

The standard deviation is presented in parentheses.

## References

[1] Fava F., Totaro G., Diels L., Reis M., Duarte J., Carioca O.B., Poggi-Varaldo H.M., Ferreira B.S. (2015). Biowaste biorefinery in Europe: Opportunities and research & development needs. New Biotechnol..

[2] Kasapidou E., Sossidou E., Mitlianga P. Fruit and vegetable processing by-/co-products: Can they be used as functional feed ingredients in animal nutrition to produce novel value-added products?. Proceedings of the 3rd International ISEKI Food Conference ISEKI_Food 2014.

[3] Chylińska M., Szymańska-Chargot M., Kruk B., Zdunek A. (2016). Study on dietary fiber by Fourier transform-infrared spectroscopy and chemometric methods. Food Chem..

[4] Nawirska A., Uklańska C. (2008). Waste products from fruit and vegetable processing as potential sources for food enrichment in dietary fibre. Acta Sci. Pol. Technol. Aliment..

[5] Nawrocka A., Miś A., Szymańska-Chargot M. (2016). Characteristics of relationships between structure of gluten proteins and dough rheology—Influence of dietary fibres studied by FT-Raman spectroscopy. Food Biophys..

[6] Shalini R., Gupta D.K. (2010). Utilization of pomace from apple processing industries: A review. J. Food Sci. Technol..

[7] Wadhwa M., Bakshi M.P.S. (2013). Utilization of Fruit and Vegetable Wastes as Livestock Feed and as Substrates for Generation of Other Value-Added Products.

[8] Balasundram N., Sundram K., Samman S. (2006). Phenolic compounds in plants and agri-industrial by-products: Antioxidant activity, occurrence, and potential uses. Food Chem..

[9] Rabetafika H.N., Bchir B., Blecker C., Richel A. (2014). Fractionation of apple by-products as source of new ingredients: Current situation and perspectives. Trends Food Sci. Technol..

[10] Dhillon G.S., Kaur S., Brar S.K. (2013). Perspective of apple processing wastes as low-cost substrates for bioproduction of high value products: A review. Renew. Sustain. Energy. Rev..

[11] Nawirska A., Kwaśniewska M. (2005). Dietary fibre fractions from fruit and vegetable processing waste. Food Chem..

[12] Szymańska-Chargot M., Cybulska J., Zdunek A. (2011). Sensing the structural differences of cellulose from apple and bacterial cell wall materials by Raman and FT-IR spectroscopy. Sensors.

[13] Taiz L., Zeiger E. (2002). Plant Physiology.

[14] Barakat A., de Vries H., Rouau X. (2013). Dry fractionation process as an important step in current and future lignocellulose biorefineries: A review. Bioresour. Technol..

[15] Arshadi M., Attard T.M., Lukasik R.M., Brncic M., Lopes A.M.D.C., Finell M., Geladi P., Gerschenson L.N., Gogus F., Herrero M. (2016). Pre-treatment and extraction techniques for recovery of added value compounds from wastes throughout the agri-food chain. Green Chem..

[16] Mosier N., Wyman C., Dale B., Elander R., Lee Y.Y., Holtzapple M., Ladisch M. (2005). Features of promising technologies for pretreatment of lignocellulosic biomass. Bioresour. Technol..

[17] Gao C., Xiao W., Ji G., Zhang Y., Cao Y., Han L. (2017). Regularity and mechanism of wheat straw properties change in ball milling process at cellular scale. Bioresour Technol..

[18] (2016). Eurostat. http://ec.europa.eu/eurostat/statistics-explained/index.php/Agricultural_products.

[19] Mirabella N., Castellani V., Sala S. (2014). Current options for the valorization of food manufacturing waste: A review. J. Clean Prod..

[20] The Food and Agriculture Organization of the United Nations (FAOSTAT) (2014). Food and Agricultural Commodities Production for, Production of Tomato by Countries. http://faostat3.fao.org/home/E.

[21] Schieber A., Stintzing F.C., Carle R. (2001). By-products of plant food processing as a source of functional compounds—Recent developments. Trends Food Sci. Technol..

[22] Kaur D., Wani A.A., Oberoi D.P.S., Sogi D. (2008). Effect of extraction conditions on lycopene extractions from tomato processing waste skin using response surface methodology. Food Chem..

[23] Yoon K.Y., Cha M., Shin S.R., Kim K.S. (2005). Enzymatic production of a soluble-fibre hydrolysate from carrot pomace and its sugar composition. Food Chem..

[24] Fonseca S.C., Oliveira F.A.R., Brecht J.K., Chau K.V., Oliveira F.A.R., Oliveira J.C. (1999). Development of perforation-mediated modified atmosphere packaging for fresh-cut vegetables. Processing Foods: Quality Optimization and Process Assessment.

[25] Tarazona-Díaz M.P., Aguayo E. (2013). Assessment of by-products from fresh-cut products for reuse as bioactive compounds. Food Sci. Technol. Int..

[26] da Silva A.E., Rodrigues Marcelino H., Salgado Gomes M.C., Oliveira E., Nagashima T., Tabosa Egito E., Verbeek J. (2012). Xylan, a promising hemicellulose for pharmaceutical use, products and applications of biopolymers. Products and Application of Biopolymers.

[27] Persin Z., Stana-Kleinschek K., Foster T.J., Van Dam J.E.G., Boeriu C.G., Navard P. (2011). Challenges and opportunities in polysaccharides research and technology: The EPNOE views for the next decade in the areas of materials, food and health care. Carbohydr. Polym..

[28] Albuquerque P.B.S., Coelho L.C.B.B., Teixeira J.A., Carneiro-da-Cunha M.G. (2016). Approaches in biotechnological applications of natural polymers. AIMS Mol. Sci..

[29] Kollarigowda R.H. (2015). Novel polysaccharide nanowires; synthesized from pectin-modified methacrylate. RSC Adv..

[30] Mierczyńska J., Cybulska J., Zdunek A. (2017). Rheological and chemical properties of pectin enriched fractions from different sources extracted with citric acid. Carbohydr. Polym..

[31] Voragen F., Beldman G., Schols H., McCleary B.V., Prosky L. (2001). Chemistry and Enzymology of Pectins. Advanced Dietary Fibre Technology.

[32] Willats W.G.T., Knox J.P., Mikkelsen J.D. (2006). Pectin: New insights into an old polymer are starting to gel. Trends Food Sci. Technol..

[33] Wang K., Jiang J.-X., Xu F., Sun R.-C., Baird M.S. (2010). Influence of steam pressure on the physicochemical properties of degraded hemicelluloses obtained from steam-exploded Lespedeza stalks. BioResources.

[34] Menon V., Prakash G., Rao M. (2010). Value added products from hemicelluloses: Biotechnological perspective. Glob. J. Biochem..

[35] Hallac B.B., Ragauska A.J. (2011). Analyzing cellulose degree of polymerization and its relevancy to cellulosic ethanol. Biofuels Bioprod. Biorefin..

[36] Shokri J., Adibkia K., van de Ven T., Godbout L. (2013). Application of Cellulose and Cellulose Derivatives in Pharmaceutical Industries. Cellulose—Medical, Pharmaceutical and Electronic Applications.

[37] Grassino A.N., Brnčić M., Vikić-Topić D., Roca S., Dent M., Brnčić S.R. (2016). Ultrasound assisted extraction and characterization of pectin from tomato waste. Food Chem..

[38] Silveira M.H.L., Morais A.R.C., Da Costa Lopes A.M., Olekszyszen D.N., Bogel-Łukasik R., Andreaus J., Pereira Ramos L. (2015). Current Pretreatment Technologies for the Development of Cellulosic Ethanol and Biorefineries. ChemSusChem.

[39] Renard C.M.G.C., Lemeunier C., Thibault J.F. (1995). Alkaline extraction of xyloglucan from depectinised apple pomace: optimization and characterisation. Carbohydr. Polym..

[40] Gírio F.M., Fonseca C., Carvalheiro F., Duarte L.C., Marques S., Bogel-Łukasik R. (2010). Hemicelluloses for fuel ethanol: A review. Bioresour. Technol..

[41] Poli A., Anzelmo G., Fiorentino G., Nicolaus B., Tommonaro G.P., Elnashar M. (2011). Di Donato, Polysaccharides from Wastes of Vegetable Industrial Processing: New Opportunities for Their Eco-Friendly Re-Use. Biotechnology of Biopolymers.

[42] Szymańska-Chargot M., Chylińska M., Farooq M. (2017). Method of Nanocellulose Preparation form Fruit Pomace, Nanocellulose Films and Method of Nanocellulose Films Preparation. Patent.

[43] Van Soest P.J. (1963). Use of detergents in the analysis of fibrous feeds. II. A rapid method for the determination of fiber and lignin. J. Assoc. Off. Agric. Chem..

[44] Szymańska-Chargot M., Zdunek A. (2013). Use of FT-IR spectra and PCA to the bulk characterization of cell wall residues of fruits and vegetables along a fraction process. Food Biophys..

[45] Szymańska-Chargot M., Chylińska M., Kruk B., Zdunek A. (2015). Combining FT-IR spectroscopy and multivariate analysis for qualitative and quantitative analysis of the cell wall composition changes during apples development. Carbohydr. Polym..

[46] Cybulska J., Zdunek A., Psonka-Antonczyk K.M., Stokke B.T. (2013). The relation of apple texture with cell wall nanostructure studied using an atomic force microscopy. Carbohydr. Polym..

[47] Montusiewicz A., Pasieczna-Patkowska S., Lebiocka M., Szaja A., Szymańska-Chargot M. (2017). Hydrodynamic cavitation of brewery spent grain diluted by wastewater. Chem. Eng. J..

[48] Renard C.M.G.C. (2005). Variability in cell wall preparations: Quantification and comparison of common methods. Carbohydr. Polym..

[49] Redgwell R.J., Curti D., Gehin-Delval C. (2008). Physicochemical properties of cell wall materials from apple, kiwifruit and tomato. Eur. Food Res. Technol..

[50] Chylińska M., Szymańska-Chargot M., Zdunek A. (2016). FT-IR and FT-Raman characterization of non-cellulosic polysaccharides fractions isolated from plant cell wall. Carbohydr. Polym..

[51] Cybulska J., Zdunek A., Kozioł A. (2015). The self-assembled network and physiological degradation of pectins in carrot cell walls. Food Hydrocoll..

[52] Albalasmeh A.A., Berhe A.A., Ghezzehei T.A. (2013). A new method for rapid determination of carbohydrate and total carbon concentrations using UV spectrophotometry. Carbohydr. Polym..

[53] Wunderlich B. (1973). Macromolecular Physic.

[54] Segal L., Creely J.J., Martin A.E., Conrad C.M. (1962). An empirical method for estimating the degree of crystallinity of native cellulose using the X-ray diffractometer. Text. Res. J..

[55] Park S., Baker J.O., Himmel M.E., Parilla P.A., Johnson D.K. (2010). Cellulose crystallinity index: Measurement techniques and their impact on interpreting cellulase performance. Biotechnol. Biofuels.

[56] Poletto M., Heitor L., Zattera A.J. (2014). Native cellulose: structure, characterization and thermal properties. Materials.

[57] Jin X.-J., Kamdem D.P. (2009). Chemical composition, crystallinity and crystallite cellulose size in Populus hybrids and aspen. Cellul. Chem. Technol..

[58] Zdunek A., Kozioł A., Pieczywek P.M., Cybulska J. (2014). Evaluation of the Nanostructure of Pectin, Hemicellulose and Cellulose in the Cell Walls of Pears of Different Texture and Firmness. Food Bioprocess Technol..

[59] Jiang F., Hsieh Y.-L. (2015). Cellulose nanocrystal isolation from tomato peels and assembled nanofibers. Carbohydr. Polym..

[60] Shulz H., Baranska M. (2007). Identification and quantification of valuable plant substances. Vib. Spectrosc..

[61] Szymańska-Chargot M., Chylińska M., Pieczywek P.M., Rösch P., Schmitt M., Popp J., Zdunek A. (2016). Raman imaging of changes in the polysaccharides distribution in the cell wall during apple fruit development and senescence. Planta.

[62] Chylińska M., Szymańska-Chargot M., Zdunek A. (2014). Imaging of polysaccharides in the tomato cell wall with Raman microspectroscopy. Plant Methods.

[63] Schenzel K., Fischer S., Brendler E. (2005). New method for determining the degree of cellulose I crystallinity by means of FT Raman spectroscopy. Cellulose.

[64] Gierlinger N., Keplinger T., Harrington M. (2012). Imaging of plan cell walls by confocal Raman microscopy. Nat. Protoc..

[65] Szymańska-Chargot M., Pieczywek P.M., Chylińska M., Zdunek A. (2016). Hyperspectral image analysis of Raman maps of plant cell walls for blind spectra characterization by nonnegative matrix factorization algorithm. Chemom. Intell. Lab. Syst..

[66] Mood S.H., Golfeshan A.H., Tabatabaei M., Jouzani G.S., Ardjmand M., Najafi G. (2013). Lignocellulosic biomass to bioethanol, a comprehensive review with focus on pretreatment. Renew. Sustain. Energy. Rev..

[67] Dufresne A. (2012). Nanocellulose: From Nature to High Performance Tailored Materials.

[68] Reddy N., Yang Y. (2005). Biofibers from agricultural byproducts for industrial applications. Trends Biotechnol..

[69] Gindl W., Thomas S., Zaikov G., Valsaraj K.T. (2009). Cellulose Fibril- and Whisker-Reinforced Polymer Nanocomposites. Recent Advances in Polymer Nanocomposites.

[70] Aditiya H.B., Mahlia T.M.I., Chong W.T., Nur H., Sebayang A.H. (2016). Second generation bioethanol production: A critical review. Renew. Sustain. Energy Rev..

[71] Khor W.C., Rabaey K., Vervaeren H. (2015). Low temperature calcium hydroxide treatment enhances anaerobic methane production from (extruded) biomass. Bioresour. Technol..

